# Machine Learning assisted systematic reviewing in orthopaedics

**DOI:** 10.1016/j.jor.2023.11.051

**Published:** 2023-11-22

**Authors:** Bart G. Pijls

**Affiliations:** Department of Orthopaedics, Leiden University Medical Center, Leiden, the Netherlands

**Keywords:** Machine learning, Artificial intelligence, Systematic review, Screening, ASReview

## Abstract

**Background:**

Machine learning assisted systematic reviewing may help to reduce the work burden in systematic reviews. The aim of this study is therefore to determine by a non-developer the performance of machine learning assisted systematic reviewing on previously published orthopaedic reviews in retrieving relevant papers.

**Methods:**

Active learning for Systematic Reviews (ASReview) was tested against the results from three previously published systematic reviews in the field of orthopaedics with 20 iterations for each review. The reviews covered easy, intermediate and advanced scenarios. The outcomes of interest were the percentage work saved at 95% recall (WSS@95), the percentage work saved at 100% recall (WSS@100) and the percentage of relevant references identified after having screened the first 10% of the records (RRF@10). Means and corresponding [95% confidence intervals] were calculated.

**Results:**

The WSS@95 was respectively 72 [71–74], 72 [72–73] and 50 [50–51] for the easy, intermediate and advanced scenarios. The WSS@100 was respectively 72 [71–73], 62 [61–63] and 37 [36–38] for the easy, intermediate and advanced scenarios. The RRF@10 was respectively 79 [78–81], 70 [69–71] and 58 [56–60] for the easy, intermediate and advanced scenarios.

**Conclusions:**

Machine learning assisted systematic reviewing was efficient in retrieving relevant papers for systematic review in orthopaedics. The majority of relevant papers were identified after screening only 10% of the papers. All relevant papers were identified after screening 30%–40% of the total papers meaning that 60%–70% of the work can potentially be saved.

## 1 Introduction

Systematic reviews and meta-analyses are an important part of evidence-based research and offer essential information for the development of clinical guidelines and decision making.^1^, ^2^, ^3^ Their strength lies in the systematic and transparent method to provide a quantitative and qualitative summary of the existing literature while minimizing the risk of bias.^1^, ^2^, ^3^

With the ever growing number of publications and increasing rate of publication, it has now become necessary for some reviews to screen more than 20.000 papers during the screening phase.^4^ Screening such large number of papers is very time consuming and may increase the risk of human error. While machine learning methods have been developed to aid in the review process, the human-in-the-loop remains necessary.^5^, ^6^, ^7^, ^8^ Therefore machine learning assisted systematic reviewing seems a viable approach and some papers have indeed suggested promising results.^7^^,^^8^ However, it is not clear how these machine learning models perform when used by non-developers and it is not clear if these results can be generalized to different disciplines such as (orthopaedic) surgery.

The aim of this study is therefore to determine by a non-developer the performance of machine learning assisted systematic reviewing on previously published orthopaedic reviews in retrieving relevant papers measured as work saved at 95% recall (WSS@95), work saved at 100% recall (WSS@100) and the percentage of relevant papers that are identified after screening 10% of the total (RRF@10).

## 2 Methods

### 2.1 Data

Three previously published systematic reviews in the field of orthopaedics were selected covering a wide range of research questions, included study designs and screening difficulty.^9^, ^10^, ^11^ The original files of the literature search and included papers were available to allow assessment of the performance of machine learning assisted systematic reviewing against the published results using conventional methods. For all reviews the inclusion rate was approximately 5%. The assigned difficulty level (easy, intermediate and advanced) was subjectively determined (by XX). The following reviews, in order of difficulty level of the screening process, were used in the present study:

### 2.2 Easy scenario

The Ribbing disease review was a systematic review on cases and case series of patients with Ribbing Disease. This review aimed to describe the clinical and radiological presentation of patients with Ribbing Disease as well as the effects of treatments that were attempted.^11^ This was a review on individual patient data. Relevant studies could be easily identified as they all mentioned Ribbing Disease or (hereditary) multiple diaphyseal sclerosis. The literature search identified 420 papers of which 23 were included in the final review.^11^

### 2.3 Intermediate scenario

The radiostereometric analysis (RSA) review was a systematic review that evaluates the migration patterns of tibial components in patients with primary total knee arthroplasty.^10^ This was a review on group level data from cohorts and randomized controlled trials (RCTs). Relevant studies could be identified (with intermediate difficulty) as they mentioned primary total knee arthroplasty, RSA, migration or similar terminology. The literature search identified 1167 papers of which 53 were included in the final review.^10^ To increase the difficulty to an intermediate level the search of 1186 papers was used that contained some doubles for the included and excluded studies.

### 2.4 Advanced scenario

Metal-on-metal (MOM) review was a systematic review that compares the mortality and morbidity of patients with MOM hip arthroplasty to patients with non-MOMhip arthroplasty.^9^ This was a review on group level data from observational studies and RCTs. The screening process was considered advanced because it requires knowledge and interpretation to correctly identify studies comparing MOM versus non-MOM. For instance, study groups were often described by the articulation of the THA such as metal-on-polyethylene or ceramic-on-ceramic for the non-MOM group and MOM or resurfacing for the MOM group. Study groups were often not identified as MOM or non-MOM in the title or abstract. Additionally, brand names of MOM implants and articulations were mentioned instead of MOM versus non-MOM further adding to the heterogeneity of used terminology. Finally, the search contained records of clinical trial registry reports (e.g. from clinicaltrials.org) which often only provide a title. All these aspects added to the complexity of the screening process.^9^ The literature search of the RCTs identified 683 papers of which 30 RCTs were included for outcome mortality.

### 2.5 Machine learning software

Active learning for Systematic Reviews (ASReview version 0.19.2; open source; https://asreview.nl/) was used for the screening phase of the reviews and the recommended default settings were used (classifier: Naïve Bayes; Query strategy max; Feature extraction: Term Frequency-Inverse Document Frequency).^8^^,^^12^ In ASReview the researcher determines which papers are relevant or not in interaction with an active machine learning model. Starting with prior knowledge of at least one relevant and one on-relevant paper, the software ranks papers by predicted relevance: the most relevant papers are shown first while the least relevant papers are shown last.^8^^,^^12^ With every decision the model updates the predicted relevancy of the remaining papers and the order in which these remaining papers are shown. This way all relevant papers could potentially be included early during the screening process thereby possibly avoiding screening of the remaining non-relevant papers.^8^^,^^12^

### 2.6 Statistics

The outcomes of interest were the percentage Work Saved (over Sampling) at 95% recall (WSS@95), the percentage Work Saved (over Sampling) at 100% recall (WSS@100) and the percentage of relevant references identified after having screened the first 10% of the records (RRF@10).^6^^,^^8^ The WSS@95 and the WSS@100 give an indication of the percentage of papers that do not need to be screened (work saved) while still finding respectively 95% or 100% of the relevant (included) papers.^6^^,^^8^ The RRF@10 gives an indication of the percentage of relevant papers that are identified after screening only a small fraction (10%) of the entire search results.^6^^,^^8^

Twenty iterations for reach systematic review were performed to calculate the mean and corresponding 95% confidence interval [95%CI] for the outcomes mentioned above.^8^^,^^12^ For each iteration one relevant and one non-relevant paper was used as prior knowledge. These papers were chosen at random. The ASReview simulation mode was used for the iterations.^8^^,^^12^ In line with recent recommendations 95% confidence intervals were calculated.^13^

## 3 Results

The data and the results from all iterations are publicly available and can be found here: osf.io/38fy5.

### 3.1 Easy scenario: the ribbing disease review

**Fig. 1:**
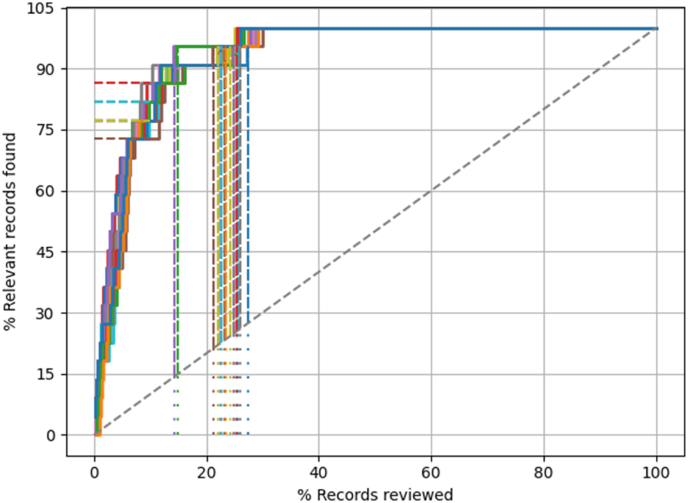
Recall curves (n = 20 iterations) of the machine learning model for finding papers of the Ribbing Disease review. The dotted diagonal line represents the rate of finding relevant papers when the papers are screened at random which is the standard way of performing systematic reviews. The dotted horizontal lines represent the proportion of relevant papers that are identified after screening 10% (RRF@10) and the dotted vertical lines show when 95% of the relevant papers have been identified (WSS@95). All relevant papers were identified after screening approximately 30% of the papers.

The result for the Ribbing disease review are presented in Table 1 and Fig. 1. The machine learning model was efficient in retrieving relevant papers. All relevant papers were identified after screening approximately 30% of the total papers, so approximately 70% of the work could be saved. When only the first 10% of papers were screened approximately 79% of all relevant paper were identified.

**Table 1:** Performance of machine learning aided systematic reviewing on orthopaedic systematic reviews.

Systematic Review	WSS@95 Mean [95%CI]	WSS@100 Mean [95%CI]	RRF@10 Mean [95%CI]
Ribbing	72 [71–74]	72 [71–73]	79 [78–81]
RSA	72 [72–73]	62 [61–63]	70 [69–71]
MOM	50 [50–51]	37 [36–38]	58 [56–60]

All means and 95% confidence intervals [95%CI] are based on 20 iterations.

WSS@95 is the percentage work saved (over sampling) at 95% recall.

WSS@100 is the percentage work saved (over sampling) at 100% recall.

RRF@10 is the percentage of relevant references identified after having screened the first 10% of the records.

RSA = radiostereometric analysis.

MOM = metal-on-metal.

### 3.2 Intermediate scenario: the RSA review

**Fig. 2:**
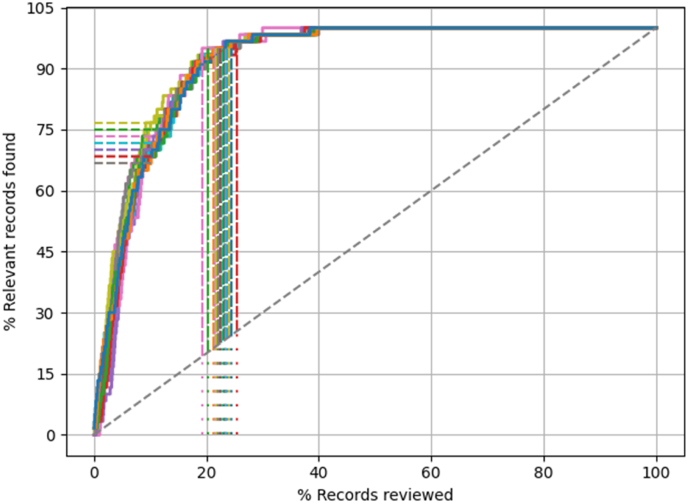
Recall curves (n = 20 iterations) of the machine learning model for finding papers of the RSA review. The dotted diagonal line represents the rate of finding relevant papers when the papers are screened at random which is the standard way of performing systematic reviews. The dotted horizontal lines represent the proportion of relevant papers that are identified after screening 10% (RRF@10) and the dotted vertical lines show when 95% of the relevant papers have been identified (WSS@95). All relevant papers were identified after screening approximately 40% of the papers.

The result for the RSA review are presented in Table 1 and Fig. 2. The machine learning model was efficient in retrieving relevant papers. All relevant papers were identified after screening approximately 40% of the total papers, so approximately 60% of the work could be saved. When only the first 10% of papers were screened approximately 70% of all relevant paper were identified.

### 3.3 Advanced scenario: MOM review

**Fig. 3:**
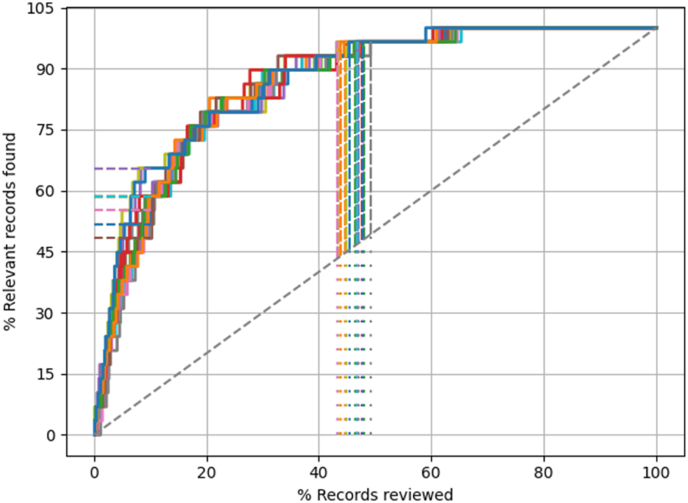
Recall curves (n = 20 iterations) of the machine learning model for finding papers of the MOM review. The dotted diagonal line represents the rate of finding relevant papers when the papers are screened at random which is the standard way of performing systematic reviews. The dotted horizontal lines represent the proportion of relevant papers that are identified after screening 10% (RRF@10) and the dotted vertical lines show when 95% of the relevant papers have been identified (WSS@95). All relevant papers were identified after screening approximately 65% of the papers.

The result for the ribbing disease review are presented in Table 1 and Fig. 3. The machine learning model was less efficient in retrieving relevant papers compared to the easy and intermediate scenarios. All relevant papers were identified after screening approximately 65% of the total papers, so approximately 35% of the work could be saved. When only the first 10% of papers were screened approximately 58% of all relevant paper were identified.

## 4 Discussion

The results of this study show that machine learning assisted systematic reviewing was efficient in retrieving relevant papers for the easy and intermediate scenarios. All relevant papers were identified after screening 30%–40% of the total papers meaning that 60%–70% of the work can potentially be saved. Compared to easy and intermediate scenarios, machine learning assisted systematic reviewing was less efficient for the advanced scenario, which required screening of 65% of the papers to find all relevant papers, so only 35% of the work could potentially be saved. The MOM review required expertise and interpretation to correctly identify studies comparing MOM versus non-MOM total hip arthroplasty, so this advanced scenario was less suitable for machine learning assisted systematic reviewing. Nevertheless, after screening only 10% of the papers, machine learning assisted systematic reviewing was able to identify the majority of relevant papers in all scenarios, which underscores the efficiency of the active machine learning model.

Our results, based on orthopaedic surgery systematic reviews, are in line with results of previous studies that tested the efficiency of the same machine learning software (ASReview).^7^^,^^8^ Van de Schoot et al. (2021) have found percentages work saved at 95% recall (WSS@95%) between 67 and 92% for non-surgical reviews which are slightly better than then 50%–72% found in the present study.^8^ Similarly, Ferdinands, (2021) has found percentages work saved at 95% recall of approximately 80% for a non-surgical review which is also slightly higher than results in the present study.^7^ The reason for these differences may be differences in disciplines e.g. surgical vs non-surgical reviews. The differences may also be due to lack of optimalization of the model in the present study as the defaults settings of the model were used. A different model, such as logistic regression, could have given better results.^7^

Some limitations should be considered. First, included papers were used to determine the performance of ASReview while relevant papers may be more appropriate for assessing the performance of ASReview during the screening phase. However, the number of relevant papers during the screening phase may vary between reviewers (experience, style of the reviewer etc.) and this was therefore considered to be a less reliable gold standard. Second, the default settings were used without optimalization of the model to the datasets. It may very well be that other settings, such as a different classifier, may yield different results. Third, only one machine learning software tool has been used. However, this tool is fully open access, all relevant documentation, data and code can be found online and it allows researchers to run the analyses on their own computer thereby being truly open science with researchers in full control.^8^^,^^12^

The following strengths could be considered. First, there was variety in difficulty of the included systematic reviews ranging from easy to advanced. Second, the study was performed by a non-developer. Third, all data and results of this study are available without restrictions at Open Science Forum.

As also suggested by others, machine learning assisted systematic reviewing could be used to reduce the workload of the reviewers during the review process even when all papers have to be screened.^5^^,^^6^^,^^8^ For instance, the screening phase could be divided into two stages: during the first stage most relevant papers are found, which requires high levels of concentration by the reviewers; followed by the second stage with few to none remaining relevant papers, which requires lower levels of concentration by the reviewers.^5^^,^^6^^,^^8^ Additionally, machine learning assisted systematic reviewing could be used in a scenario where a second (senior) researcher could screen and identify most of the relevant papers by screening only the first stage of the screening phase, instead of screening a random set of papers.^5^^,^^6^^,^^8^ The challenge is to correctly identify where the first stage ends and the second stage begins during the screening process. From a clinical perspective machine learning assisted systematic reviewing could be used by clinicians to quickly find most (all) relevant papers on a subject to guide clinical decision making. This gives orthopaedic surgeons the opportunity to find relevant studies themselves. Machine learning assisted systematic reviewing could make them less dependent on third party (prescription based) evidence-based summaries and it also allows them to check these summaries.

Future studies could further focus on the possible association between difficulty of the review and the work saved. This would allow researchers to estimate a-priori how much of the screening needs to be done using machine learning models in order to find (almost) all relevant papers.

In conclusion machine learning assisted systematic reviewing was efficient in retrieving relevant papers for systematic review in orthopaedics. The majority of relevant papers were identified after screening only 10% of the papers.

## Ethical statement

This work involves meta-data from prior systematic reviews and does not involve individual patient data. Hence informed consent was not necessary.

## Funding statement

There was no external funding for this work.

## Guardian/patients consent

This work involves meta-data from prior systematic reviews and does not involve individual patient data. Hence patient consent was not necessary.

## CRediT authorship contribution statement

**Bart G. Pijls:** Conceptualization, Methodology, Data curation, Writing – original draft, Visualization, Investigation, Writing – review & editing.

## Declaration of competing interest

None.
